# Nurse Managers' Perspectives on Digital Transformation and Informatics Competencies in E-Leadership: A Qualitative Study

**DOI:** 10.1155/jonm/8178924

**Published:** 2025-07-21

**Authors:** Ebtsam Abou Hashish, Sharifa Alsayed, Badriah Hassan Mohammed Alqarni, Nouf Majdou Alammari, Rahaf Obedallah Alsulami

**Affiliations:** ^1^College of Nursing-Jeddah, King Saud bin Abdul-Aziz University for Health Sciences, Jeddah, Saudi Arabia; ^2^King Abdullah International Medical Research Center, Jeddah, Saudi Arabia; ^3^Faculty of Nursing, Alexandria University, Alexandria, Egypt

**Keywords:** e-leadership, health information management, hospitals, information literacy, leadership, nurse administrators

## Abstract

**Aim:** To explore nurse managers' perspectives on the competencies required for effective e-leadership in the context of healthcare digital transformation.

**Background:** As healthcare systems adopt digital technologies, nurse managers must acquire informatics and leadership competencies to navigate evolving responsibilities, improve team performance, and enhance care quality.

**Methods:** A qualitative descriptive design was used at a major Saudi hospital. Semistructured interviews were conducted with a purposive sample of 42 nurse managers between 2023 and 2024. Data were analyzed thematically using Braun and Clarke's six-step approach.

**Results:** Participants were predominantly female (90.5%) and Saudi nationals (52.4%), with most aged 40–50 years. While all held at least a bachelor's degree, 61.9% had a master's qualification. Most had 5–10 years of managerial experience, though 61.9% reported lacking digital leadership training. Thematic analysis identified six key themes: digital transformation, e-leadership, e-leadership competencies (knowledge, skills, and attitudes), self-efficacy evaluation, barriers, and recommendations. Participants recognized the transformative role of digital tools but cited challenges such as limited training, infrastructure constraints, and hierarchical resistance.

**Conclusion:** Nurse managers acknowledged the need for enhanced digital literacy, technical proficiency, and a positive attitude toward change. Strengthening these competencies is essential for leading digital innovation and supporting healthcare reform initiatives such as Saudi Arabia's Vision 2030.

**Implications for Nursing Management:** Healthcare organizations should invest in structured leadership development, mentorship, and academic-practice partnerships to equip nurse managers with the skills and confidence to lead digital transformation effectively.

## 1. Background

Over the past 2 decades, digital transformation has rapidly reshaped various industries, including healthcare [1, 2]. This process integrates technology into all business areas to enhance efficiency and scalability [3, 4]. In healthcare, digital transformation has given rise to digital healthcare, improving health outcomes and addressing global challenges such as infectious and chronic diseases [5, 6]. Digital tools now reduce the need for in-person consultations and hospitalizations and increasingly influence nursing practices worldwide [7–10].

Digital transformation in nursing and healthcare is marked by the rise of AI, robotic systems, and the expanded use of telehealth, especially in response to the COVID-19 pandemic [7]. Healthcare systems are increasingly adopting hospital information systems, telemedicine, electronic medical records (EMRs), and remote monitoring technologies, supported by smartphone apps and decision-support tools [11, 12]. Likewise, in education, digital tools such as e-learning, virtual reality, and game-based learning are enhancing professional development [13, 14]. Moreover, personalized healthcare is being advanced through assistive devices, sensors, and ambient-assisted living technologies [7, 10].

Nursing and healthcare leadership are on the brink of a significant transformation [15]. The rapid advancement of digital technology presents both opportunities and challenges for hospital administrators and nursing leaders [16, 17]. Nursing leaders, such as directors, supervisors, and unit managers, oversee multiple units or programs within healthcare systems, requiring continuous communication with frontline staff, administrative support, and other leaders. As the healthcare sector evolves, care delivery increasingly incorporates virtual teams and clinics. Consequently, nurse leaders are relying more on digital communication tools, such as email, instant messaging, video conferencing, and web chats, to lead their teams, particularly when physical presence in the clinical environment is not feasible [18]. This shift has given rise to the concept of “e-leadership,” where leaders engage with their teams through technology to disseminate and gather information [19, 20].

Specifically, nurse leaders have complex role profiles that include direct responsibility for leading nursing units or a team of staff nurses, executing daily information technology coordination tasks (e.g., email communication, data review, and decision-making) [21], and appearing to be more involved in the implementation and management of digital health services [22, 23]. As a result, they are becoming “e-leaders,” utilizing a variety of information technologies to achieve various objectives. E-leadership is seen as a potent social influence technique in which information technologies can impact attitudes, feelings, thinking, behavior, and performance [24].

### 1.1. E-Leadership Framework for Digital Transformation

A recent review by Cortellazzo et al. [24] emphasized that studies exploring the impact of digital transformation on leadership often lack a guiding theoretical model. To address this gap, the current study adopted Avolio et al.'s [25] e-leadership framework, which conceptualizes leadership in digitally mediated environments through four interrelated mechanisms: characteristics, cognition, affect, and behavior. This framework provides a holistic view of how leaders operate and influence others in virtual or hybrid contexts, making it particularly suitable for examining nurse managers' roles in digitally transforming healthcare systems.

Characteristics refer to the personal qualities, values, and identity of the leader, essentially, who one is. In nursing management, this includes a leader's integrity, adaptability, confidence in using technology, and commitment to innovation. These traits influence how nurse managers present themselves as digital role models and shape their credibility in leading transformation. Cognition involves the mental processes leaders use, what and how they think, in making decisions, analyzing digital information, and solving problems in virtual settings. For nurse managers, this includes interpreting dashboards, evaluating informatics reports, and applying critical thinking in technology-enabled decision-making. Affect captures the emotional tone and interpersonal sensitivity of lead*ers,* what one feels. In digital environments, nurse managers' ability to convey empathy, reassurance, or urgency through mediated communication (e.g., emails and chat systems) affects team morale and engagement. Behavior reflects what the leader does, including how they use digital platforms, coordinate virtual tasks, model new technology use, and shape team interactions. For nurse managers, this encompasses promoting the use of hospital systems, encouraging staff training, and troubleshooting digital workflows [25]. Applying this model may enable a deeper understanding of how nurse managers navigate their leadership roles within a digital healthcare environment, especially within the context of Saudi Arabia's Vision 2030 and its emphasis on digital health innovation.

### 1.2. Significance of the Study

The accelerating digital transformation in healthcare has fundamentally reshaped the role of nurse leaders. As healthcare systems adopt electronic health records, clinical decision-support systems, telehealth platforms, and AI-driven analytics, nurse managers are increasingly expected to lead teams in digitally enabled environments [7, 26]. Their responsibilities have expanded from traditional administrative oversight to strategic digital leadership, including the management of informatics workflows and the development of staff digital competencies [27].

Globally, several studies have explored facets of this transition. For example, Laukka et al. [18, 28], in Finland, investigated nurse leaders' involvement in digital health service implementation and their informatics-related competencies. Bleijenbergh et al. [29] used a Delphi approach in Belgium to identify digital adaptability competencies among healthcare professionals but did not focus on nursing leadership. Similarly, Schiuma et al. [30] analyzed transformative leadership competencies for hospital digital transformation, yet their focus remained at the executive level rather than on frontline nurse managers.

In Saudi Arabia, digital transformation is a cornerstone of Vision 2030, which emphasizes enhanced healthcare efficiency, transparency, and innovation. National strategies led by the Ministry of Health include the adoption of e-health systems, artificial intelligence, and virtual care platforms [15]. Despite these reforms, there remains limited empirical evidence on how nurse managers, key frontline leaders, perceive their own digital readiness, role expectations, and informatics competencies [15, 31]. Recent studies in the Gulf region have primarily focused on the digital literacy of staff nurses or on infrastructural readiness, with little attention to the lived experiences and leadership challenges of nurse managers during digital transformation.

For instance, Zakaria et al. [15] highlighted fragmented career pathways and inadequate leadership training in Saudi Arabia's digital health workforce. AlYami et al. [31] assessed nurses' digital preparedness but did not address managerial competencies or leadership roles. While these findings underscore growing awareness of digital capacity-building in nursing, they largely overlook the practical and emotional realities that nurse managers face as digital change agents.

Moreover, limited existing studies have applied a structured theoretical framework, such as Avolio et al.'s [25] e-leadership model, to examine how nurse managers conceptualize and perform digital leadership. This model, which encompasses leader characteristics, cognition, affect, and behavior, offers a valuable lens to understand leadership in technology-mediated environments. Its absence from empirical studies on nurse managers presents a critical gap in both research and practice. Therefore, this study addresses that gap by exploring how nurse managers in a Saudi tertiary hospital experience and evaluate their e-leadership roles and informatics competencies using a qualitative design. Findings are expected to inform future leadership development, curriculum design, and workforce strategies to support Saudi Arabia's national digital health agenda through confident and competent nurse leadership.

### 1.3. Aim of the Study

This study aimed to explore nurse managers' perspectives on the competencies required for their e-leadership roles in the context of digital transformation.

## 2. Methods

### 2.1. Study Design and Setting

An exploratory qualitative research design was employed at King Khalid Hospital (KKH), Jeddah, Saudi Arabia. This qualitative descriptive approach is effective for gathering in-depth, direct, and firsthand accounts of phenomena that have limited existing information. It is particularly well-suited for exploring and understanding a phenomenon, process, or the perspectives of participants [32]. Consequently, this approach was chosen in the present study to thoroughly explore nurse managers' perspectives on the competencies necessary for their e-leadership roles. The study was reported in accordance with the Consolidated Criteria for Reporting Qualitative Research (COREQ) checklist.

### 2.2. Study Subjects and Sampling

The study utilized purposive sampling to include frontline nurse leaders or nurse managers as participants (*N* = 42). Eligibility criteria required participants to be employed at KKH, have experience in providing digital health services (such as electronic health records, patient portals, and online communication platforms), hold a position as a frontline nurse leader or manager, and consent to participate in the current study. The exclusion criteria were bedside nurses and nurse interns. The sample size was determined by the principle of data saturation, defined as the point at which no new information emerged [33].

### 2.3. Data Collection Instrument

Semistructured interviews (SSI): The interview guide, developed by the researchers, was designed to gather the necessary information. To ensure its validity, the guide was reviewed by the researchers' peers, who unanimously agreed on its content. Two pilot interviews were conducted to verify the clarity of the questions, refine them as needed, and ensure the researchers were comfortable with the interview technique before the main data collection phase. After this validation process, the data collection began.

The interview guide consisted of two sections as follows:

• Section 1: Demographic characteristics: This section included questions about age, gender, unit, nationality, digital tools, or apps used in the work area and previous training on digital tools, forms, or digital leadership.
• Section 2: Open-ended questions: This section featured overarching questions designed to elicit qualitative data and allow participants to express their perspectives on digital leadership and competencies. This study was guided by Avolio et al.'s [25] e-leadership framework, which conceptualizes leadership in technology-mediated environments through four key mechanisms: characteristics, cognition, affect, and behavior. The following research questions guided the interview:
  1. What do you know about digital transformation in healthcare?
  2. What do you know about digital health service leadership (e-leadership)?
  3. What digital competencies do nurse managers require in their e-leadership roles?
  4. How do you assess your own competencies as a nurse or nursing manager?
  5. What are the barriers to becoming an effective e-leader, and what recommendations do you have for overcoming them?

For example:

• The question “What do you know about digital transformation?” and “What digital competencies are required?” captured participants' knowledge and understanding (cognition).
• The question “How do you assess your own competencies?” explored participants' self-concept and leadership identity (characteristics).
• The question “What are the barriers to becoming an effective e-leader, and what recommendations do you have for overcoming them?” explored emotional dimensions through responses to barriers and adaptation challenges (affect).
• Questions about strategies and leadership actions, such as “What recommendations do you have to become an effective e-leader?,” captured observable behaviors and practices (behavior).

While the interview guide was informed by Avolio's model, the thematic analysis was conducted inductively by using Braun and Clarke's [34] six-phase framework, allowing themes to emerge organically from the data.

### 2.4. Data Collection and Ethical Considerations

The researchers interviewed the willing participants after obtaining the approval from King Abdullah International Medical Research Center (KAIMRC) (IRB: SP23J/131/08) and hospital approvals. These interviews were conducted in English at times and in places convenient for the participants. The researchers informed the participants about the study's nature, the ethical considerations involving human subjects, the estimated duration of the interview, data confidentiality, voluntary participation, informed consent, their right to withdraw from the study, and the use of quoted information at the start of each interview. To ensure anonymity in the presentation of interview excerpts, the researchers assigned participants numbers instead of names. Each interview lasted between 30 and 45 min, and all interviews were recorded with detailed notes taken immediately after each session. The researchers transcribed the interviews with participants' permission prior to data analysis. The data collection period spanned six months, from November 2023 to April 2024.

### 2.5. Data Analysis

Data collection and analysis were conducted concurrently, beginning after the first interview and continuing throughout the data collection period. Demographic data were analyzed descriptively using frequencies and percentages. For qualitative data, the researchers applied thematic analysis following the six-phase approach developed by Braun and Clarke [34], which offers a systematic and flexible method for identifying patterns in qualitative data.

The process began with familiarization, during which all audio-recorded interviews were transcribed verbatim and reviewed repeatedly by the research team to gain a deep understanding of the content and context. In the second phase, initial coding, two researchers independently coded the transcripts line-by-line. Key phrases and significant segments were manually highlighted and labeled using Microsoft Excel, which was used to organize and manage the data.

In the theme development phase, similar codes were clustered into conceptual groups to form subthemes based on semantic similarities and shared meaning. Subthemes that reflected broader underlying ideas were then synthesized into overarching themes. The criteria for defining a major theme included the recurrence of a concept across multiple interviews, its relevance to the study aim, and its conceptual richness. This process was iterative, involving repeated refinement and reorganization of categories to ensure internal consistency and distinction between themes.

To ensure clarity and consistency in naming, the research team prioritized using participant language and culturally meaningful expressions where applicable. Each theme and subtheme was clearly defined in terms of its scope and meaning. Team discussions were held to validate the relevance and boundaries of themes, and refinements were made until consensus was reached. Representative quotes were selected to illustrate each subtheme, strengthening the link between participant narratives and the analytic structure. In the fourth and fifth phases, reviewing and defining themes, the team ensured that each theme was coherent and adequately captured the coded data. To enhance credibility, member checking was performed with a subset of participants to verify that the themes accurately reflected their views. In addition, peer debriefing among the research team supported reflexivity and reduced individual bias.

**Figure 1:**
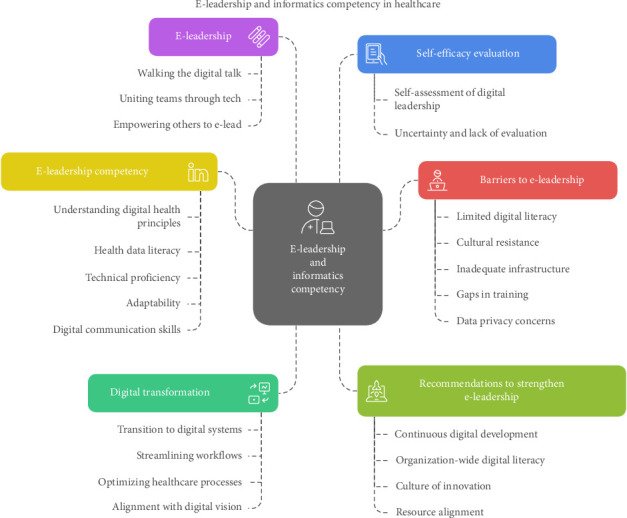
Emerged themes regarding digital transformation and e-leadership from managerial perspectives.

Finally, in the reporting phase, the findings were organized into a table outlining the six major themes and subcategories (Table 1) and visualized through Figure 1. Although Avolio et al.'s [25] e-leadership framework informed the design of the interview guide and supported interpretation in the discussion, the themes were derived inductively from the data and not preimposed by the framework. This ensured that the analysis remained grounded in participants' lived experiences.

### 2.6. Trustworthiness and Rigor

To ensure trustworthiness and rigor, the researchers focused on credibility, transferability, dependability, and confirmability [34]. For credibility, they developed participant selection criteria and an interview guide, regularly reviewed transcripts, and performed member checking to validate the findings with participants, who suggested no changes. Transferability was achieved by providing a detailed description of the methods and data in the final report. Dependability was ensured through consistency checks by a peer researcher, and confirmability was maintained through regular peer debriefing sessions and an audit trail. Direct quotes from interviewees were used to support the findings and link them to the data.

## 3. Results

### 3.1. Participants' Characteristics

A total of 42 nurse managers were interviewed, the majority of whom were female (90.5%) and Saudi nationals (52.4%). Most participants were aged 40–50 years and held at least a bachelor's degree, with 61.9% having a master's qualification. While 40.5% had over 20 years of nursing experience, the majority (81%) had 5–10 years in managerial roles. Notably, while 38.1% (*n* = 16) have received training on digital tools or leadership, 61.9% (*n* = 26) lacked such training. Participants worked across diverse clinical units and reported frequent use of digital systems such as Best Care, Oracle, attendance platforms, Safety Reporting System (SRS), Information Services Division (ISD), and Microsoft Office tools, mainly to enhance communication, administrative efficiency, and patient safety (see Table 2).

### 3.2. Participant's Perspectives on E-Leadership and Informatics Competencies

#### 3.2.1. Theme Derivation Process

Thematic analysis resulted in six major themes, each comprising multiple subcategories that were inductively derived through line-by-line coding of participants' transcripts. These subcategories reflect recurring patterns, perspectives, and processes described by nurse managers. The analysis was conducted following Braun and Clarke's framework, ensuring a systematic approach to identifying and refining themes based on conceptual similarity rather than predefined categories.

The key themes that emerged include digital transformation, e-leadership, e-leadership competencies (encompassing knowledge, skills, and attitudes), self-efficacy and evaluation of e-leadership, barriers to effective e-leadership, and recommendations to strengthen e-leadership. Each theme is supported by a set of clearly defined subcategories that capture the depth and diversity of participants' experiences. Statements from participants were anonymized and coded using identifiers such as P#1, P#2, and so on, reflecting the order in which interviews were conducted. Table 1 presents a summary of the themes, their corresponding subcategories, and illustrative participant quotations that exemplify the core meaning of each category.

#### 3.2.2. Theme I: Digital Transformation

This theme captures nurse managers' collective understanding of digital transformation as a progressive, systemwide shift from manual, paper-based processes to integrated electronic platforms. Participants described how this transition encompassed the adoption of tools such as EMRs, Oracle-based HR systems, and communication applications such as CTS, email, WhatsApp, and Best Care. They highlighted that these systems contributed to improved workflow standardization, interdisciplinary collaboration, and tracking of staff performance and clinical documentation. Beyond operational benefits, nurse managers framed digital transformation as a strategic priority, aligning with Saudi Arabia's Vision 2030. They perceived it not only as a technological advancement but as a national mandate driving quality, safety, and modernization in healthcare delivery.

Sample quotation:

“Digital transformation in healthcare is moving us from manual to electronic and online systems. We've eliminated paper records at the bedside, transitioning to electronic systems for tasks such as medication safety and staff competency assessments.” P# 9

#### 3.2.3. Theme II: E-Leadership

This theme reflects nurse managers' understanding of their evolving leadership role in supporting digital transformation. E-leadership was described not merely as the use of digital tools but as a behavioral and relational role that involves setting an example, fostering a supportive team culture, and enabling others to confidently navigate digital systems.

Participants viewed leading by example, what some called “walking the digital talk,” as a core leadership strategy. By actively using systems such as Best Care, Oracle, and digital documentation tools, nurse managers sought to model expected behaviors and demonstrate the value of digital integration in everyday practice. Several emphasized that staff adoption often depended on the leader's own visibility and confidence in using these systems.

Beyond personal competence, nurse managers also saw their role as creating a collaborative environment. where digital adoption is supported through team-based learning. Encouraging peer support, facilitating digital knowledge-sharing, and addressing staff resistance were seen as critical components of successful e-leadership. Furthermore, many participants highlighted the importance of building future digital leaders by empowering others, encouraging innovation, and providing guidance.

Sample quotation:

“We encourage staff to support each other on digital tasks, no one should be left behind.” P#18

#### 3.2.4. Theme III: E-Leadership Competencies

This theme reflects the range of competencies that nurse managers consider essential for effective leadership in a digitally transforming healthcare environment. Participants identified three interconnected domains, knowledge, skills, and attitudes, that together form the foundation of successful e-leadership. These are collectively referred to as the e-leadership competency triangle, emphasizing the balance needed between what nurse leaders understand, do, and believe in order to guide digital transformation successfully (see Table 1).

##### 3.2.4.1. Knowledge

Nurse managers emphasized the need for comprehensive knowledge of digital systems and strong data and information literacy. This included understanding the principles and applications of digital health technologies, along with the ability to interpret, manage, and communicate healthcare data. Such competencies were seen as essential for driving improvements in care and decision-making.

Sample quotation:

“We must have strong data skills to turn information into actions that improve patient care and efficiency.” P#14

##### 3.2.4.2. Skills

Participants emphasized the importance of hands-on technical proficiency and confidence when using tools such as Best Care and CTS. Equally important were the ability to adapt to new technologies, advocate for digital adoption, make evidence-informed decisions, and communicate clearly while leading teams through digital change. These skills were described as practical, developmental, and essential for motivating others.

Sample quotation:

“I think we need basic computer skills, confidence with technology, and an open mind to learn and adapt to new digital tools.” P#1

##### 3.2.4.3. Attitudes

The emotional and cognitive orientation of nurse managers toward digital transformation was seen as equally vital. Most expressed openness and optimism, acknowledging that digitalization is an inevitable and necessary evolution in healthcare. However, participants also acknowledged concerns, including system usability and fear of errors. Attitudes reflected both confidence and caution, pointing to the need for training, support, and a growth-oriented mindset.

Sample quotation:

“I am excited and proud about the implementation of digital services in healthcare.” P#15

#### 3.2.5. Theme IV: Self-Efficacy and Evaluation of E-Leadership

This theme explores how nurse managers assess their own competencies and confidence in leading digital transformation. Participants described a wide range of perceived self-efficacy, from moderate competence to strong digital leadership behaviors, as well as uncertainty about how to evaluate their digital readiness.

##### 3.2.5.1. Self-Evaluation of Digital Leadership Efficacy

Several participants provided reflective assessments of their digital leadership abilities, often rating themselves as moderately competent or “in the middle.” Some emphasized that while they were not digital experts, they felt capable of navigating new systems and supporting their teams. Others expressed confidence in their leadership role, noting their ability to model positive digital attitudes and facilitate staff adaptation.

Sample quotation:

“I'd say I'm moderately competent in digital transformation, may be a 6 out of 10. It's still pretty new to me.” P#17

##### 3.2.5.2. Uncertainty and Limited Self-Assessment

Other participants expressed a lack of clarity in evaluating their digital leadership skills, either because of insufficient feedback mechanisms or limited opportunities for formal training and development. These nurse managers emphasized the need for structured self-assessment tools, digital leadership benchmarks, and institutional support to build confidence.

Sample quotation:

“Honestly, I haven't had much experience evaluating my own skills in digital transformation. But I'm planning to start some training sessions for my team to get everyone up to speed.” P#40

#### 3.2.6. Theme V: Barriers to Becoming an Effective E-Leader

Nurse managers identified a range of barriers that hinder their ability to lead effectively in digital healthcare environments. A central concern was limited digital literacy and user confidence. Several participants acknowledged struggling with digital tools, which made it difficult to model technology use or support staff in digital adoption efforts. This lack of confidence often led to dependence on more technologically skilled colleagues. Another major barrier involved resistance to digital change, both at the individual and organizational levels. Nurse managers shared that some team members were hesitant or unwilling to use new systems, which slowed the adoption process. In addition, some described a workplace culture that did not prioritize or support digital innovation, making leadership efforts more difficult.

Technical limitations and inadequate infrastructure were also frequently cited. Participants described challenges such as limited access to computers, unstable internet connections, and outdated software or systems that interfered with daily operations and team collaboration. These issues reduced the credibility of digital initiatives and disrupted key leadership activities such as virtual meetings and documentation.

In parallel, many managers reported training gaps and a lack of structured support for developing their digital leadership roles. While expectations were high, few had received formal education or mentoring on how to lead effectively in a digital healthcare environment.

Lastly, data privacy and system reliability were significant concerns. Participants were cautious about embracing digital transformation fully due to fears of compromising patient data or encountering system crashes during critical tasks.

Sample quotation:

“Sometimes workplace culture doesn't always support new technology, making it hard to lead digitally.” P#29

#### 3.2.7. Theme VI: Recommendations to Strengthen E-Leadership

Nurse managers outlined several strategies to overcome barriers and promote effective e-leadership in the digital transformation era. A central recommendation was the implementation of continuous digital development and feedback mechanisms. Participants stressed the need for ongoing training programs, regular updates on technological advances, and support systems that enable leaders to adapt over time. They also valued opportunities for self-paced learning and professional development to sustain competence.

Sample quotation:

“We need regular updates and support programs to keep improving.” P#1

Managers emphasized the importance of promoting organizationwide digital literacy, noting that digital education should not be limited to leaders. Inclusive training that accounts for varying levels of digital fluency was seen as essential to build a confident, digitally capable workforce across all levels of care.

Sample quotation:

“Digital education should be for everyone, not just managers.” P#34

To create an environment conducive to digital change, participants highlighted the need to cultivate a culture of innovation and team support. Encouraging staff participation in decision-making, recognizing digital contributions, and allowing space for experimentation were considered important motivators. Nurse managers called for a shift in mindset, where digital efforts are celebrated rather than penalized.

Sample quotation:

“Involving staff in decision-making processes can help increase buy-in and reduce resistance.” P#30

Finally, nurse managers advocated for resource alignment and improved technical infrastructure. They pointed to the need for adequate hardware, stable internet access, and financial resources to support both technology upgrades and training delivery. Reliable systems were seen not just as logistical necessities but as enablers of effective leadership.

Sample quotation:

“Resources must match the expectations, we need working systems and access.” P#32

## 4. Discussion

The literature highlights the critical need to prepare a health management workforce capable of navigating the complexities of digital health, emphasizing the importance of equipping nurse managers to lead effectively in this domain [35]. This study provides valuable insights into nurse managers' evolving roles in digital healthcare leadership. By exploring their experiences and perspectives, the study identified six interrelated themes that reflect both personal competencies and systemic influences. The findings underscore the transformative impact of digitalization on leadership practices, while also highlighting contextual nuances shaped by Saudi Arabia's Vision 2030. The findings are discussed as follows, organized by the main themes starting with digital transformation.

### 4.1. Digital Transformation (Theme I)

Nurse managers in this study viewed digital transformation as more than a technical upgrade; it signaled a fundamental shift in leadership, communication, and decision-making practices. Digital systems such as EMRs, Oracle, and Best Care were seen as enabling more streamlined workflows and cross-functional collaboration.

This interpretation is consistent with global literature. In Australia, Brommeyer et al. [35] emphasized the need to prepare healthcare managers to lead digital innovation. In Saudi Arabia, Abou Hashish and Alnajjar [8] called for aligning digital competencies with Vision 2030. Similar views have emerged internationally: Reis et al. [3], in Latin America, described digital transformation as restructuring healthcare delivery; Mauro et al. [36], in the United States, noted its role in modernizing patient care; Staras et al. [37], in the United States, found improvements in workflow efficiency; and Bhardwaj et al. [38], in Indi, linked digitalization to care quality. In Nordic countries, Laukka et al. [28], in Finland, and Oksavik et al. [39], in Norway, reported that digital tools supported workflow redesign and goal alignment, despite challenges in relational leadership.

In Saudi Arabia, Vision 2030 prioritizes digital transformation through national initiatives such as “Seha Virtual Hospital and EMR expansion” [40]. However, our findings suggest that without empowering nurse leaders at the operational level, such initiatives risk remaining superficial. Similar to recommendations by the Nordic Healthcare Group [41], parallel investments in leadership capacity are necessary. This is especially important in hierarchical systems such as Saudi Arabia's, where nurse managers require structured training and mentorship to translate national strategy into actionable change [42].

### 4.2. E-Leadership (Theme II)

Nurse managers in this study described e-leadership as a dynamic approach requiring digital fluency, virtual team collaboration, and a mindset geared toward innovation and continuous learning. This marks a shift from traditional hierarchical leadership toward participatory and digitally integrated models. This perspective aligns with Avolio et al. [19], who defined e-leadership as influencing others via advanced technologies, combining technical competence with virtual trust-building. Abuowda et al. [43] also emphasized balancing digital skills with emotional intelligence. Liu et al. [44] outlined a progressive model of e-leadership, from basic digital communication to full integration of leadership and technology, while Van Wart et al. [45] stressed blending electronic and traditional communication strategies. Cortellazzo et al. [24] underscored the adaptive nature of digital leadership in transformation contexts.

Nurse managers in our study echoed these global insights but reported constraints in authority, preparation, and support, challenges intensified in Saudi Arabia's hierarchical healthcare structures. While Vision 2030 promotes digital transformation, its success depends on equipping nurse leaders to actively drive change at the frontline. This finding mirrors those from Finland and Norway, where digital enthusiasm among nurse leaders is often not matched by formal training or decision-making autonomy [28, 39]. The Nordic Healthcare Group (2023) argues that aligning leadership development with digital strategy is essential. In Saudi Arabia, building e-leadership capacity, through structured education, mentorship, and empowerment, is especially critical to translating national digital goals into everyday clinical practice [9, 42].

### 4.3. E-Leadership Competencies (Theme III)

Nurse managers in this study consistently emphasized three interconnected domains, knowledge, skills, and attitudes, critical for effective digital leadership. Inductively, these were conceptualized as a competency triangle, aligning with Avolio et al.'s [25] framework, where cognition, affect, and behavior represent core dimensions of e-leadership.

Knowledge was seen as foundational. Participants highlighted the importance of understanding digital transformation principles, data systems, and communication technologies. They stressed the need to interpret healthcare data accurately and apply it strategically. These views resonate with Schiuma et al. [30], who emphasized digital dexterity and strategic planning as essential leadership competencies. Similar emphasis on informatics literacy and cognitive readiness is found in the works of Abou Hashish and Alnajjar [8], the WHO [26], and Farias-Gaytan et al. [46]. Andrade [47] and Wilandika et al. [48] further supported this perspective by linking data literacy with improved operational efficiency and patient outcomes. In the Saudi context, informatics knowledge remains an underdeveloped area among nurse leaders [42].

In terms of skills, nurse managers identified technical proficiency, digital self-efficacy, adaptability, and evidence-based decision-making as crucial. They described competencies in using EMRs, Oracle, and other platforms, while also emphasizing leadership in change management and team support. These findings align with Laukka et al. [28], who identified visionary leadership and informatics proficiency as critical skills. Avolio et al. [25], Laukka et al. [18], and Pawar and Dhumal [49] emphasized the value of leveraging AI and data analytics for agile leadership. Cortellazzo et al. [24] and Laukka et al. [28] reinforced the importance of virtual communication and remote leadership capabilities, especially in digitally mediated work environments.

Attitude emerged as equally essential. Participants expressed varying degrees of enthusiasm and apprehension, but overall, recognized the need for a positive, open-minded approach to digital transformation. This supports findings by Rajamani et al. [50] and Stoumpos et al. [51], who noted that optimism about digital change fosters innovation and performance. Booth [7] and Laukka et al. [18] similarly argued that nurse leaders play a pivotal role in shaping attitudes and promoting digital engagement within teams. Finally, participants emphasized the importance of recognizing team readiness and individual digital competencies. Laukka et al. [18, 52] also highlighted the role of nurse leaders in identifying “digital champions” to facilitate adoption and co-create workflows that align with clinical realities.

In summary, the competency triangle, knowledge, skills, and attitudes, offers a holistic view of what is required for effective e-leadership. Aligned with Avolio's model, these findings underscore that successful nurse leaders must combine digital literacy, emotional readiness, and strategic behavior to guide meaningful and sustainable digital transformation.

### 4.4. Self-Efficacy and Evaluation of E-Leadership Competencies (Theme IV)

Nurse managers in this study reflected critically on their own digital leadership capabilities, revealing a spectrum of self-efficacy. While some reported moderate confidence and readiness to guide digital initiatives, others expressed uncertainty, particularly around technical proficiency and digital system navigation. These differences were largely shaped by disparities in training, system exposure, and organizational support.

Many described themselves as being “in-between,” neither novices nor experts, signaling an emerging self-awareness of their evolving leadership roles. This informal process of self-assessment acted as a catalyst for identifying growth areas and adapting to digital expectations. These findings correspond with Avolio et al.'s [25] framework, which suggests that cognitive and affective self-perceptions influence e-leadership behavior. Leaders' confidence in their digital abilities directly impacted how they engaged with teams and technology. This reflective orientation aligns with Isidori et al. [53] and Abou Hashish et al. [54], who advocate for leadership development models that integrate technical skill-building, communication, and self-awareness. Similarly, Laukka et al. [28] found that nurse leaders often develop digital competencies through informal, self-directed learning rather than structured training, reinforcing the value of mentorship and feedback loops.

In the Saudi context, where Vision 2030 is driving rapid digital reform, self-efficacy becomes a pivotal element of sustainable e-leadership. However, national strategies must be matched by practical, culturally attuned interventions. Structured professional development, regular feedback, and mentorship programs, tailored to the realities of hierarchical institutions, are essential to empower nurse managers to lead confidently and effectively [30].

### 4.5. Barriers to E-Leadership (Theme V)

Despite the promise of digital transformation, nurse managers identified five interrelated barriers that hinder effective e-leadership: digital literacy gaps, organizational resistance, infrastructure and resource limitations, training deficiencies, and ethical concerns. Limited digital literacy emerged as a persistent challenge for both nurse managers and their teams, leading to reliance on IT support and reduced leadership confidence. This aligns with Gkrimpizi et al. [55] and Laukka et al. [18], who documented similar deficits globally. In Saudi Arabia, public hospitals face uneven access to digital health tools, contributing to disparities in digital readiness [56].

Organizational resistance was another key barrier. Participants described hierarchical structures, reluctance from senior leaders, and skepticism among staff as obstacles to digital adoption. These concerns reflect findings by Scholkmann [57] and Gjellebæk et al. [16], and are reinforced in the Saudi context, where centralized governance often limits nurse leaders' decision-making authority [58]. Infrastructure and resource constraints were also widely reported. Inadequate devices, unstable internet, outdated software, and limited funding hindered digital progress, challenges similarly noted by Mumtaz et al. [59] and Kaihlanen et al. [60]. These issues obstruct the behavioral enactment of e-leadership, as outlined in Avolio et al. [25] model.

Training gaps remain a critical concern. Many managers lacked formal preparation and relied on informal learning, echoing global trends reported by Laukka et al. [28]. While Saudi Arabia's Vision 2030 emphasizes digital reform, implementation has often outpaced leadership capacity-building [8, 31]. In addition, ethical concerns, particularly around data privacy, contributed to hesitancy. Participants expressed fears about mishandling patient information in unfamiliar systems. This aligns with Abuzaid et al. [61] and Strudwick et al. [27], who stress the ethical burden of digital leadership and the need for robust governance structures.

Although Saudi Arabia is well-resourced, a strategic gap persists in preparing nurse leaders for the digital era. Qtait and Jaradat [62] noted not only systemic issues such as workforce instability, gender disparities, and resistance to change but also highlighted opportunities for leadership education and policy reform. Mani and Goniewicz [63] emphasized that national workforce development efforts must be complemented by investments in digital leadership training.

### 4.6. Recommendations to Strengthen E-Leadership (Theme VI)

To overcome the barriers identified in this study, nurse managers recommended several strategies, which were grouped into four core areas: continuous digital development, systemwide training, cultural transformation, and resource alignment.

First, participants emphasized the need for continuous professional development and structured feedback. They advocated for hands-on, self-paced digital training, regular system updates, and ongoing mentorship to sustain competency and confidence. These strategies echo international recommendations; Laukka et al. [18] and Konttila et al. [64], in Finland, highlighted formal informatics education as essential for innovation leadership. Similarly, Abernethy et al. [65] in the United Kingdom emphasized the integration of ethical governance into leadership training. In the Arab context, Qtait and Jaradat [62] advocated for digital learning platforms and mentorship to build leadership capacity and system resilience.

Second, participants called for broad-based digital literacy initiatives that extend beyond nurse managers to include all healthcare staff. This view is supported by Schiuma et al. [30], who emphasized that fostering a culture of digital fluency at all levels is critical to transformation success.

Third, the importance of cultivating a supportive, innovation-driven culture was repeatedly stressed. Nurse managers recommended inclusive decision-making, recognition of digital efforts, and experimentation as mechanisms to boost morale and ownership. These views align with findings by Mumtaz et al. [59] in Pakistan, who emphasized proactive leadership as key to overcoming change resistance. In Saudi Arabia, Vision 2030 provides a national mandate for digital transformation, but participants highlighted the need for greater authority and structured training to enact these roles effectively. International comparisons show that in countries such as Canada, the Netherlands, and Nordic nations, nurse leaders are more involved in strategic digital decision-making [41, 51]. In contrast, Saudi nurse managers operate within more hierarchical systems, requiring tailored empowerment strategies that reflect the local context.

Fourth, adequate infrastructure and aligned resources were considered prerequisites for effective digital leadership. Participants cited the need for updated systems, reliable internet, and sufficient hardware. Borges do Nascimento et al. [66], in Brazil, and Gkrimpizi et al. [55], in Greece, similarly stressed that infrastructure quality directly affects digital adoption. In addition, participants emphasized the importance of data security and privacy training, concerns mirrored in Canadian and Finnish studies [18, 27], which called for comprehensive data governance frameworks.

These recommendations suggest that strengthening e-leadership requires more than individual skill-building; it involves systemic reform. For Saudi Arabia, aligning such strategies with Vision 2030 objectives and adapting them to the country's hierarchical and multicultural healthcare dynamics will be key to sustaining digital transformation and optimizing leadership impact.

### 4.7. Contextualizing E-Leadership Within Saudi Arabia's Vision 2030 Framework

This study highlights the need for culturally tailored leadership development aligned with Saudi Arabia's Vision 2030 and the day-to-day realities of nurse managers. Vision 2030 prioritizes digital health through major initiatives such as Seha Virtual Hospital, EMR expansion, and national telehealth integration [67]. However, strong policy direction has not translated into consistent implementation due to systemic and cultural barriers.

Nurse managers reported operating within centralized, hierarchical structures where decision-making is top-down, limiting innovation and autonomy. The workforce's diversity and dependence on expatriate professionals contribute to inconsistent digital fluency and access to training [67, 68]. Cultural norms such as deference to authority and resistance to change also impact e-leadership readiness [63].

These factors influence the knowledge, skills, and attitudes that make up the e-leadership competency triangle and help explain observed readiness gaps. Achieving Vision 2030's digital goals requires not only technological investment but also decentralization, inclusive training, and culturally adapted leadership strategies [63].

## 5. Strengths and Limitations

This study provides a robust and contextually grounded examination of the competencies required for nurse managers to effectively lead digital transformation within healthcare settings. It highlights not only the technical and leadership skills needed but also organizational and cultural factors that influence e-leadership in practice. A major strength of the study lies in its thematic depth, capturing a wide range of challenges, development needs, and strategic recommendations derived from nurse managers lived experiences. In addition, the study introduces a competency triangle framework (knowledge, skills, and attitudes) aligned with Avolio's e-leadership model, offering a practical lens for understanding leadership in digitally evolving systems. The focus on self-evaluation, continuous development, and system-level recommendations strengthens its contribution to leadership capacity-building initiatives, particularly in alignment with Saudi Arabia's Vision 2030 digital health agenda.

Nonetheless, the study has certain limitations. It was conducted within a single tertiary hospital in Saudi Arabia, which may limit the generalizability of the findings to other institutional or national contexts with different technological infrastructures, workforce compositions, or governance models. Furthermore, the qualitative data were based on individual accounts, which may be influenced by organizational dynamics or social desirability. While the study captured diverse managerial roles and experiences, it did not include objective measures of digital leadership competencies. Despite these limitations, the findings provide a timely and actionable foundation for advancing digital leadership in nursing management.

## 6. Conclusion

This study highlights the critical role of nurse managers in leading digital transformation within healthcare settings and offers a comprehensive framework for understanding the competencies required for effective e-leadership. Through an in-depth qualitative analysis, the study identified six key themes that reflect the evolving demands placed on nurse managers in the digital era, ranging from digital transformation awareness to leadership capabilities, self-assessment, systemic challenges, and practical strategies for improvement.

By introducing the e-leadership competency triangle, encompassing knowledge, skills, and attitudes, and aligning it with Avolio's e-leadership framework, the study contributes a theoretically grounded and practice-informed model that can guide leadership development. The findings also contextualize these needs within the Saudi healthcare system and Vision 2030, illustrating how national-level ambitions must be matched with localized, empowered leadership development strategies.

## 7. Implications of the Study

### 7.1. Implications for Practice

Healthcare organizations, particularly in rapidly transforming systems such as Saudi Arabia's, should use the identified competencies to design structured and culturally aligned training programs. Emphasizing the e-leadership competency triangle, knowledge, skills, and attitudes, can guide leadership development efforts in both technical and behavioral domains. Nurse managers should be empowered to lead digital initiatives by advocating for resource allocation, facilitating interprofessional collaboration, and integrating evidence-based digital practices. These actions are vital to overcoming resistance, fostering innovation, and improving care quality in digital healthcare environments.

### 7.2. Implications for Nursing Management

For nursing management, the study underscores the need for leadership commitment to a culture of continuous digital learning. Nurse managers must engage in ongoing professional development to remain responsive to technological change, regulatory demands, and patient needs. In hierarchical and diverse systems such as Saudi Arabia's, management should also invest in mentorship, inclusive leadership structures, and shared decision-making. Building stronger partnerships with IT departments and involving nurses in the design and deployment of digital systems will ensure frontline engagement and system usability. The findings also suggest that leadership development should be embedded in national digital health strategies such as Vision 2030.

### 7.3. Implications for Healthcare Systems

At the health system level, integrating e-leadership competencies into organizational strategy can advance national reform goals and enhance systemwide performance. Robust digital infrastructure alone is insufficient without competent leadership to guide its application. By embedding data-driven decision-making, telehealth integration, and AI-informed leadership into operational workflows, nurse leaders can enhance patient safety, care personalization, and organizational responsiveness.

### 7.4. Implications for Future Research

Building on this study, future research should focus on developing and validating a structured assessment tool for e-leadership competencies tailored to nurse managers. This tool should reflect the competency triangle and themes explored, knowledge, skills, attitudes, self-evaluation, barriers, and recommendations. Longitudinal and multicenter studies in Saudi Arabia and other healthcare systems will be essential to evaluate the tool's impact on leadership effectiveness and patient outcomes. In addition, future work should explore the influence of cultural factors, such as hierarchy, language diversity, and gender dynamics, on digital leadership practices. Evaluating the effectiveness of interventions (e.g., leadership academies, digital mentorship, or AI-enhanced training platforms) will also be critical for scaling sustainable, context-responsive e-leadership models globally.

## Figures and Tables

**Table 1:** Emerged themes and categories and representative participant quotations.

Themes	Categories	Representative participant quotation
I. Digital transformation	1. Transition to digital systems and tools	*“We are now expected to use Oracle, attendance, and Best Care in all daily routines.” P#12*
	2. Streamlining workflows and collaboration	*“Digital systems have made coordination between units easier and faster.” P#6*
	3. Optimizing healthcare processes	*“Things are more accurate now, especially with patient records and medication timing.” P#14*
	4. Alignment with Saudi digital vision	*“All of this is part of Vision 2030, we are moving toward full digital healthcare.” P#1*

II. E-leadership	5. Walking the digital talk	*“If I don't use the system myself, my staff won't follow, leadership starts with action.” P#21*
	6. Uniting teams through tech	*“Digital leadership is about making sure the whole team grows together.” P#30*
	7. Empowering others to E-lead	*“I try to show them it's not just about tech, it's about growth and leadership.” P#6*

III. E-leadership competency	8. Understanding digital health principles and services	*“Understanding and using healthcare data are essential for us. We need to accurately analyze data, use data tools, keep data secure, and share insights effectively.” P#7*
	9. Health data and information literacy	
	10. Technical proficiency and digital self-efficacy	
	11. Adaptability and advocacy for digital change	*“Change is hard, but I keep pushing myself and others to keep up.” P#6*
	12. Evidence-based digital decision-making	*“From my perspective, we should use the best evidence to assess digital systems and make informed decisions to improve patient outcomes.” P#12*
	13. Leadership and digital communication skills	*“I believe we need skills in change management, emotional intelligence, critical thinking, and evidence-based practice to navigate digital healthcare.” P#6*
	14. Openness and optimism toward digital change	
	15. Recognizing inevitability and future readiness	*“I see digital transformation as an inevitable change now and in the future, but we need more time and support to adjust.” P#34*
	16. Mixed emotions: hope, concern, and uncertainty	*“Sometimes, system issues can disrupt our work when we're trying to care for patients.” P#34*

IV. Self-efficacy evaluation of e- leadership	17. Self-assessment of digital leadership efficacy	*“I make sure to show my team how going digital can make their jobs easier.” P#26*
	18. Uncertainty and lack of structured evaluation	*“Honestly, I haven't had much experience evaluating my own skills in digital transformation.” P#40*

V. Barriers to becoming an effective e-leader	19. Limited digital literacy and user confidence	*“Many of us struggle with using digital tools effectively and lack digital literacy, which makes it harder to lead online.” P#27*
	20. Cultural resistance and leadership disconnect	*“Some team members resist new digital technologies, slowing down our ability to improve how we work.” P#15*
	21. Inadequate infrastructure and technical support	*“Limited resources are a major barrier. Not having enough access to the right technology limits how well we can use digital tools.” P#32*
	22. Gaps in training and digital role preparation	*“We were never trained for this, we had to figure it out ourselves.” P#29*
	23. Concerns about data privacy and reliability	*“Data privacy and security concerns are real challenge… Managing patient data safely online is a big concern.” P#29*

VI. Recommendations to strengthen e-leadership	24. Continuous digital development and feedback	*“Digital education should be for everyone, not just managers.” P#34*
	25. Organizationwide digital literacy	*“We need a workplace that encourages innovation, not punishes mistakes.” P#28*
	26. Culture of innovation and team support	*“Resources must match the expectations, we need working systems and access.” P#32*
	27. Resource alignment and access	*“We need faster internet and technical support, it affects care.” P#41*

**Table 2:** Demographic characteristics and digital context of nurse manager participants (*N* = 42).

Demographic variable	Category	Frequency (*n*)	Percentage (%)
Gender	Female	38	90.5
	Male	4	9.5

Nationality	Saudi	22	52.4
	Non-Saudi	20	47.6

Age group	40–< 50 years	30	71.4
	30–< 40 years	8	19.0
	20–< 30 years	4	9.5

Education level	Bachelor's degree	16	38.1
	Master's degree	26	61.9

Years of nursing experience	Over 20 years	17	40.5
	11–15 years	8	19.0
	6–10 years	9	21.4
	1–5 years	8	19.0

Years of managerial experience	5–10 years	34	81.0
	Less than 5 years	8	19.0

Previous training on digital tools/leadership	Yes	16	38.1
	No	26	61.9

Most common work units	ICU	10	23.8
	Surgical and medical wards	12	28.6
	Obstetrics/labor and delivery	6	14.3
	Dialysis, oncology, cardiac, other	14	33.3

Most commonly used digital tools/apps	Best Care System	42	100.0
	Oracle, attendance, SRS, ECTS, and ISD	30	71.4
	Microsoft Office Suite	25	59.5
	Philips monitors, Johnson & Johnson (JJ) website, and MEOS, others	18	42.9

## Data Availability

The data that support the findings of this study are available from the corresponding author upon reasonable request.

## References

[1] Marwaha J. S., Raza M. M., Kvedar J. C. (2023). The Digital Transformation of Surgery. *NPJ Digital Medicine*.

[2] Reis J., Melão N. (2023). Digital Transformation: A Meta-Review and Guidelines for Future Research. *Heliyon*.

[3] Reis J., Amorim M., Melão N., Matos P., Rocha Á., Adeli H., Reis L. P., Costanzo S. (2018). Digital Transformation: a Literature Review and Guidelines for Future Research. *Trends and Advances in Information Systems and Technologies. WorldCIST’18 2018. Advances in Intelligent Systems and Computing*.

[4] Abdolkhani R., Petersen S., Walter R., Zhao L., Butler-Henderson K., Livesay K. (2022). The Impact of Digital Health Transformation Driven by COVID-19 on Nursing Practice: Systematic Literature Review. *JMIR Nursing*.

[5] Nes A., Steindal S., Larsen M., Heer H. C., Lærum-Onsager E., Gjevjon E. R. (2021). Technological Literacy in Nursing Education: a Scoping Review. *Journal of Professional Nursing*.

[6] Werner L., Puta C., Chilalika T. (2023). How Digital Transformation Can Accelerate Data Use in Health Systems. *Frontiers in Public Health*.

[7] Booth R. G., Strudwick G., McBride S., O’Connor S., Solano López A. L. (2021). How the Nursing Profession Should Adapt for a Digital Future. *BMJ*.

[8] Abou Hashish E. A., Alnajjar H. (2024). Digital Proficiency: Assessing Knowledge, Attitudes, and Skills in Digital Transformation, Health Literacy, and Artificial Intelligence Among University Nursing Students. *BMC Medical Education*.

[9] Abou Hashish E. A. (2025). Compassion Through Technology: Digital Empathy Concept Analysis and Implications in Nursing. *Digital health*.

[10] Atalla A. D. G., El-Gawad Mousa M. A., Abou Hashish E. A., Elseesy N. A. M., Abd El kader Mohamed A. I., Sobhi Mohamed S. M. (2025). Embracing Artificial Intelligence in Nursing: Exploring the Relationship Between Artificial intelligence-related Attitudes, Creative self-efficacy, and Clinical Reasoning Competency Among Nurses. *BMC Nursing*.

[11] Alipour J., Mehdipour Y., Karimi A., Khorashadizadeh M., Akbarpour M. (2023). Security, Confidentiality, Privacy and Patient Safety in the Hospital Information Systems From the Users’ Perspective: a cross-sectional Study. *International Journal of Medical Informatics*.

[12] Thapa S., Nielsen J. B., Aldahmash A. M., Qadri F. R., Leppin A. (2021). Willingness to Use Digital Health Tools in Patient Care Among Health Care Professionals and Students at a University Hospital in Saudi Arabia: Quantitative cross-sectional Survey. *JMIR Medical Education*.

[13] Zhang L., Wu J., Yang J. (2023). Development and Application Evaluation of a Nursing Simulation Teaching Information System Based on Hospital Information Systems. *International Journal of Clinical Practice*.

[14] Abou Hashish E. A., Al Najjar H., Alharbi M., Alotaibi M., Alqahtany M. M. (2024). Faculty and Students’ Perspectives Towards Game-based Learning in Health Sciences Higher Education. *Heliyon*.

[15] Zakaria N., Zakaria N., Alnobani O. (2023). Unlocking the Ehealth Professionals’ Career Pathways: a Case of Gulf Cooperation Council Countries. *International Journal of Medical Informatics*.

[16] Gjellebæk C., Svensson A., Bjørkquist C., Fladeby N., Grundén K. (2020). Management Challenges for Future Digitalization of Healthcare Services. *Futures*.

[17] Fletcher M., Read C., D-Adderio L. (2023). Nurse Leadership post-COVID Pandemic: a Framework for Digital Healthcare Innovation and Transformation. *SAGE Open Nursing*.

[18] Laukka E., Hammarén M., Pölkki T., Kanste O. (2023). Hospital Nurse Leaders’ Experiences with Digital Technologies: a Qualitative Descriptive Study. *Journal of Advanced Nursing*.

[19] Avolio B., Kahai S., Dodge G. (2000). E-leadership: Implications for Theory, Research, and Practice. *The Leadership Quarterly*.

[20] Numanovic V., Jalonen H., Lindell J., Jacobsson J. (2024). E-leadership in Nursing–A Systematic Review. *Finnish Journal of eHealth and eWelfare*.

[21] Cowan L. D. (2014). E-leadership Leading in a Virtual Environment—Guiding Principles for Nurses. *Nursing Economics*.

[22] Keijser W., Smits J., Penterman L., Wilderom C. (2016). Physician Leadership in e-health? A Systematic Literature Review. *Leadership in Health Services*.

[23] Elsayed W., Sleem W. (2020). Nurse Managers’ Perceptions and Attitudes Toward Using Artificial Intelligence Technology in Nursing Settings. *Assiut Scientific Nursing Journal*.

[24] Cortellazzo L., Bruni E., Zampieri R. (2019). The Role of Leadership in a Digitalized World: a Review. *Frontiers in Psychology*.

[25] Avolio B. J., Sosik J. J., Kahai S. S., Baker B. (2014). E-Leadership: Re-Examining Transformations in Leadership Source and Transmission. *The Leadership Quarterly*.

[26] World Health Organization (WHO) (2019). WHO Guideline: Recommendations on Digital Interventions for Health System Strengthening. https://apps.who.int/iris/handle/10665/311941.

[27] Strudwick G., Nagle L., Kassam I., Pahwa M., Sequeira L. (2019). Informatics Competencies for Nurse Leaders: a Scoping Review. *The Journal of Nursing Administration: The Journal of Nursing Administration*.

[28] Laukka E., Pölkki T., Kanste O. (2022). Leadership in the Context of Digital Health Services: a Concept Analysis. *Journal of Nursing Management*.

[29] Bleijenbergh R., Mestdagh E., Timmermans O., Van Rompaey B., Kuipers Y. J. (2023). Digital Adaptability Competency for Healthcare Professionals: a Modified Explorative e-Delphi Study. *Nurse Education in Practice*.

[30] Schiuma G., Santarsiero F., Carlucci D., Jarrar Y. (2024). Transformative Leadership Competencies for Organizational Digital Transformation. *Business Horizons*.

[31] AlYami A., Majrashi N., Hazazi L. (2024). Assessment of Undergraduates Nursing Students’ Knowledge Toward MRI Safety: Cross-Sectional Study. *Journal of Radiation Research and Applied Sciences*.

[32] Bradshaw C., Atkinson S., Doody O. (2017). Employing a Qualitative Description Approach in Health Care Research. *Global Qualitative Nursing Research*.

[33] Saunders B., Sim J., Kingstone T. (2018). Saturation in Qualitative Research: Exploring Its Conceptualization and Operationalization. *Quality and Quantity*.

[34] Braun V., Clarke V. (2006). Using Thematic Analysis in Psychology. *Qualitative Research in Psychology*.

[35] Brommeyer M., Liang Z. (2022). A Systematic Approach in Developing Management Workforce Readiness for Digital Health Transformation in Healthcare. *International Journal of Environmental Research and Public Health*.

[36] Mauro M., Noto G., Prenestini A., Sarto F. (2024). Digital Transformation in Healthcare: Assessing the Role of Digital Technologies for Managerial Support Processes. *Technological Forecasting and Social Change*.

[37] Staras S., Tauscher J. S., Rich N. (2021). Using a Clinical Workflow Analysis to Enhance Ehealth Implementation Planning: Tutorial and Case Study. *JMIR mHealth and uHealth*.

[38] Bhardwaj A., Patel V., Kumar R. (2021). Impact of Digital Transformation on Healthcare Quality and Efficiency. *Health Care Management Review*.

[39] Oksavik J. D., Vik E., Kirchhoff R. (2024). Digital Leadership: Norwegian Healthcare Managers’ Attitudes Towards Using Digital Tools. *Digital health*.

[40] Suleiman A. K., Ming L. C. (2025). Transforming Healthcare: Saudi Arabia’s Vision 2030 Healthcare Model. *Journal of Pharmaceutical Policy and Practice*.

[41] Gustafsson C., Dannapfel P. (2024). Leaders’ Experiences of Successfully Implementing Health and Welfare Technology in Sparsely Populated Nordic Areas. *Disability and Rehabilitation: Assistive Technology*.

[42] Alsadaan N., Ramadan O. M. E. (2025). Barriers and Facilitators in Implementing Evidence-based Practice: a Parallel cross-sectional Mixed Methods Study Among Nursing Administrators. *BMC Nursing*.

[43] Abuowda A., Iwidat H., Alawnah M. (2024). Impact of E-leadership on Organizational Citizenship Behaviour of Faculty Members in Higher Education: Information and Communication Technology as a Mediator. *Discover Education*.

[44] Liu C., Van Wart M., Kim S., Wang X., McCarthy A., Ready D. (2020). The Effects of National Cultures on Two Technologically Advanced Countries: the Case of e-leadership in South Korea and the United States. *Australian Journal of Public Administration*.

[45] Van Wart M., Roman A., Wang X., Liu C. (2019). Operationalizing the Definition of E-Leadership: Identifying the Elements of E-Leadership. *International Review of Administrative Sciences*.

[46] Farias-Gaytan S., Aguaded I., Ramirez-Montoya M. S. (2023). Digital Transformation and Digital Literacy in the Context of Complexity Within Higher Education Institutions: A Systematic Literature Review. *Humanities and Social Sciences Communications*.

[47] Andrade C. (2021). The Inconvenient Truth About Convenience and Purposive Samples. *Indian Journal of Psychological Medicine*.

[48] Wilandika A., Pandin M. G. R., Yusuf A. (2023). The Roles of Nurses in Supporting Health Literacy: A Scoping Review. *Frontiers in Public Health*.

[49] Pawar S., Dhumal V. (2024). The Role of Technology in Transforming Leadership Management Practices. *Multidisciplinary Reviews*.

[50] Rajamani S., Hultman G., Bakker C., Melton G. B. (2022). The Role of Organizational Culture in Health Information Technology Implementations: A Scoping Review. *Learning Health Systems*.

[51] Stoumpos A. I., Kitsios F., Talias M. A. (2023). Digital Transformation in Healthcare: Technology Acceptance and Its Applications. *International Journal of Environmental Research and Public Health*.

[52] Laukka E., Huhtakangas M., Heponiemi T., Kanste O. (2020). Identifying the Roles of Healthcare Leaders in HIT Implementation: a Scoping Review of the Quantitative and Qualitative Evidence. *International Journal of Environmental Research and Public Health*.

[53] Isidori V., Diamanti F., Gios L. (2022). Digital Technologies and the Role of Healthcare Professionals: Scoping Review Exploring Nurses’ Skills in the Digital Era and in the Light of the COVID-19 Pandemic. *JMIR Nursing*.

[54] Abou Hashish E. A., Khattab S. M. A. K., Mohammad H. F., Elliethey N. S. (2025). Exploring the Impact of Leadership, Organizational Support, and Knowledge Management in Evidence-based Practice Implementation Among Nurse Managers Through SEM. *Worldviews on Evidence-Based Nursing*.

[55] Gkrimpizi T., Peristeras V., Magnisalis I. (2023). Classification of Barriers to Digital Transformation in Higher Education Institutions: Systematic Literature Review. *Education Sciences*.

[56] Al-Anezi F. M. (2025). Challenges of Healthcare Systems in Saudi Arabia to Delivering Vision 2030: an Empirical Study from Healthcare Workers Perspectives. *Journal of Healthcare Leadership*.

[57] Scholkmann A. B. (2020). Resistance to (Digital) Change: Individual, Systemic, and learning-related Perspectives. *Digital Transformation of Learning Organizations*.

[58] Alenezi I. N. (2023). Leadership Experiences of Nurse Managers in a Saudi Ministry of Health Hospital: a Focused Ethnographic Study. *Research Square Preprint*.

[59] Mumtaz H., Riaz M. H., Wajid H. (2023). Current Challenges and Potential Solutions to the Use of Digital Health Technologies in Evidence Generation: A Narrative Review. *Frontiers in Digital Health*.

[60] Kaihlanen A. M., Gluschkoff K., Laukka E., Heponiemi T. (2021). The Information System Stress, Informatics Competence and well-being of Newly Graduated and Experienced Nurses: A cross-sectional Study. *BMC Health Services Research*.

[61] Abuzaid M. M., Elshami W., Fadden S. M. (2022). Integration of Artificial Intelligence into Nursing Practice. *Health Technology*.

[62] Qtait M., Jaradat Y. (2025). Nursing Leadership in the Arab World: Challenges and Opportunities for Success. *AG Salud*.

[63] Mani Z. A., Goniewicz K. (2024). Transforming Healthcare in Saudi Arabia: A Comprehensive Evaluation of Vision 2030’s Impact. *Sustainability*.

[64] Konttila J., Siira H., Kyngäs H. (2019). Healthcare Professionals’ Competence in Digitalisation: A Systematic Review. *Journal of Clinical Nursing*.

[65] Abernethy A., Adams L., Barrett M. (2022). The Promise of Digital Health: Then, Now, and the Future. *Discussion Paper*.

[66] Borges do Nascimento I. J., Abdulazeem H., Vasanthan L. T. (2023). Barriers and Facilitators to Utilizing Digital Health Technologies by Healthcare Professionals. *npj Digital Medicine*.

[67] (2030). Saudi Vision. https://www.vision2030.gov.sa/en/.

[68] Al-Hanawi M. K., Khan S. A., Al-Borie H. M. (2019). Healthcare Human Resource Development in Saudi Arabia: Emerging Challenges and Opportunities—A Critical Review. *Public Health Reviews*.

[69] Elsheikh A. S., Alqurashi A. M., Wahba M. A., Hodhod T. E. (2018). Healthcare Workforce in Saudi Arabia Under Saudi Vision 2030. *Journal of Health Informatics in Developing Countries*.

